# Prevalence of fascioliasis (liver flukes) infection in cattle and buffaloes slaughtered at the municipal abattoir of El-Kharga, Egypt

**DOI:** 10.14202/vetworld.2017.914-917

**Published:** 2017-08-13

**Authors:** Nagwa T. Elshraway, Wafaa G. Mahmoud

**Affiliations:** 1 Department of Food Hygiene, Faculty of Veterinary Medicine (New Valley), Assiut University, Assiut, Egypt; 2 Department of Parasitology, Faculty of Veterinary Medicine (New Valley), Assiut University, Assiut, Egypt

**Keywords:** *Fasciola gigantica*, *Fasciola hepatica*, foodborne disease, liver fluke, slaughterhouse, snails, zoonosis

## Abstract

**Aim:**

The main objectives of this study were to determine the prevalence of fascioliasis infections in cattle and buffaloes, slaughtered in El-Kharga city slaughterhouse at New Valley Governorate.

**Materials and Methods:**

The slaughtered animals were daily inspected for liver fascioliasis allover 2016. Macroscopic fascioliasis was detected from a total of 2251 basing on animals specie, sex, season, and *Fasciola* spp. in addition to microscopic examination of blood, fecal samples which collected from female cattle and buffalo (50 each).

**Results:**

The total prevalence rate of *Fasciola* sp. infection occurs in the study area were about 695/2251 (30.88%) from the total cattle and bovine slaughtered carcasses. The incidence of fascioliasis was 4/12 (33.33%) and 678/2200 (30.82%) for females and males cattle carcasses, respectively, while the infection rate in buffalo carcasses was 1/4 (25.00%) and 12/35 (34.29%) for females and males buffalo carcasses, respectively.

**Conclusion:**

The moderate fasciolosis infection in cattle and buffaloes slaughtered at the municipal abattoir of El-Kharga, Egypt. The highest fascioliasis infection was recorded during winter and autumn. It constitutes a major cause of economic losses at El-Kharga abattoir and threat public health.

## Introduction

Slaughterhouses provide an excellent meat inspection place where many zoonotic diseases observed but meat poor handling in or out the abattoir can leading to both economic losses and a lot of public health hazardous [1,2].

Fascioliasis considered the top of all the domestic ruminants’ parasitic zoonotic worldwide infection that is endemic in a tropical area and Egypt [3-5]. Genus *Fasciola* “liver fluke” is belonging to trematode helminths which containing two main species; *Fasciola gigantic* and *Fasciola hepatica* in Egypt [6-8].

Fascioliasis reduces animal productivity, weight gain, and the production of meat and milk. In addition, it causes moderate icterus, metabolic disorders, and secondary infections due to decrease immunity by chronic fascioliasis and liver condemnation during postmortem inspection in slaughterhouses while the acute fascioliasis may lead to mortalities [9-11].

Human fascioliasis infection occurs accidentally after ingestion of eggs/larvae while ruminant ingestion of forage containing metacercarial cyst [12]. Ingested parasite lives in hepatic parenchyma or in bile duct, which causing liver hemorrhagic black tunnels [13].

Diagnosis is depend on the history of snail habitats or fascioliasis on the farm, symptoms, postmortem examinations, feces, and blood examination for *Fasciola* eggs [14]. There is not enough information on the ruminants’ fascioliasis in El-Kharga, New Valley Governorate, Egypt. Therefore, this study was designed with the aims of determining the prevalence of fascioliasis infections in cattle and buffaloes, slaughtered in El-Kharga city slaughterhouse at New Valley Governorate.

## Materials and Methods

### Ethical approval

This study has been approved by the Animal Rights and Ethical Use Committee of Assiut University.

### The study area

A cross-sectional study was conducted in El-Kharga abattoirs to detect the prevalence of the fascioliasis (liver flukes) slaughtered cattle. El-Kharga city is the capital of New Valley Governorate. It is a part of the oasis, which is located to the west of the Nile Valley between 25.26°N latitude and 30.32°E longitude. New Valley Governorate is located 232 km to the South of Assiut Governorate and represented it is about 45% from the total Egypt area. El-Kharga abattoir slaughtered about 2200 and 12 (bulls and caws) cattle, respectively, and 35 and 4 (bulls and caws) bovine animals during 2016. According to the Egyptian legislations of meat inspection, slaughtering of cattle, and bovines female never been allowed before all teeth are changed (over 5 years) while male bovines and cattle approved for slaughtering after about 2 years.

### Samples collection

A total of 2212 (2200 bulls and 12 caws) local breed cattle and 39 (35 male and 4 females) local breed buffaloes, slaughtered at El-Kharga abattoir, were daily inspected for the presence of liver fascioliasis allover 2016 which efficiently inspected by naked eye and palpation for the presence of gross lesion and the worms then further examinations. All data samples recorded before transported in an icebox to the laboratory to the Central Laboratory, Faculty of Veterinary Medicine, New Valley, Assiut University, for further examinations within 24 h.

### Samples preparation for postmortem inspection

Liver and gall bladder postmortem inspection by making multiple cuts and subcuts about 1 cm thick to check the presence of fascioliasis, which made gritty sounds and bile duct thickness, palpation pressure, exerted brownish fluid, and immature *Fasciola*. Identification of the species based on the morphological features of the agent and classify into *F. gigantic* and *F. hepatica* [15,16].

To calculate the total sample size, the following assumptions were made: 5% desired level of precision, 95% level of confidence, and 60% expected the prevalence of cattle fascioliasis in El-Kharga abattoirs, the sample size was determined using the formula given below [17].


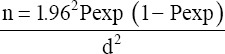


n=Required sample size, Pexp=Expected prevalence, d=Desired absolute precision.

Therefore, based on the above formula, the total sample size of cattle was calculated to be 2.80.

### Statistical analysis

The obtained results were encoded and recorded in an excel database analyzed by descriptive statistics survey were performed using GraphPad Instant version 3 for determination of means and the analysis of variance between the different data. The treatment, in this study, was determined using standard error and analysis of variance (p<0.05).

## Results

### Prevalence of liver fascioliasis in examined cattle and buffaloes samples

The results obtained in Table 1 indicated that the total prevalence rate of *Fasciola* sp. infection occurs in the study area were about 695/2251 (30.88%) from the total cattle and bovine slaughtered carcasses. The incidence of fascioliasis was 4/12 (33.33%) and 678/2200 (30.82%) for females and males cattle carcasses, respectively, while the infection rate in buffalo carcasses was 1/4 (25.00%) and 12/35 (34.29%) for females and males buffalo carcasses, respectively.

**Table-1:** Prevalence of liver fascioliasis in examined cattle and buffaloes slaughtered at the municipal abattoir of El-Kharga.

Examined animals	Examined	Positive	Prevalence %
Cattle			
Females	12	4	33.33
Males	2200	678	30.82
Buffaloes			
Females	4	1	25.00
Males	35	12	34.29
Total	2251	695	30.88

### Seasonal liver fascioliasis condemnation rates in examined cattle and buffaloes samples

**Figure-1:**
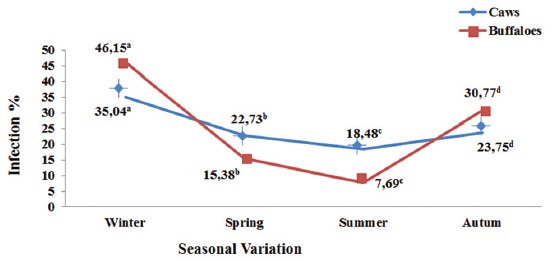
Seasonal liver fascioliasis condemnation rates in examined cattle and buffaloes slaughtered at the municipal abattoir of El-Kharga. Means followed by a different letter in the line are significantly different (p>0.05).

As illustrated in Figure-1, results revealed that buffaloes fascioliasis is higher during winter and autumn than cattle fascioliasis while vice versa condition reported during spring and summer. The highest fascioliasis infection found in winter followed by autumn, spring, and summer. There was a significant difference in between different seasons while there was not any significance between caws and buffaloes rates.

The buffaloes rates were (46.15%, 15.38%, 7.69%, and 30.77%) during winter, spring, summer and autumn, respectively, whereas in case of cattle, it were (35.04%, 22.73%, 18.48%, and 23.75%) during winter, spring, summer, and autumn, respectively.

### Macroscopic liver fascioliasis in examined cattle and buffaloes samples

**Figure-2:**
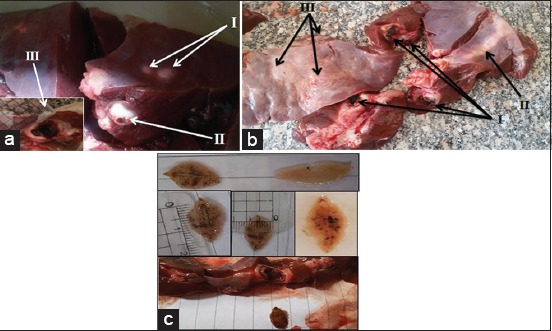
Macroscopic liver fascioliasis detected from cattle, and bovine carcasses in examined cattle and buffaloes slaughtered at the municipal abattoir of El-Kharga. (a) I - Varies large sizes (1.5-2.7 cm) fascioliasis tunnels observed on intact external liver surfaces, II - Liver postmortem slicing showing fibrosis fascioliasis tunnels, and III - Cut section of fascioliasis tunnels, its sizes about (1.5 cm) observed infected liver. (b) I - Several liver fascioliasis tunnels oozing grassy blackish hemorrhagic exudate varies sizes (0.5-1.5 cm), II - Fascioliasis tunnels observed on intact external liver surfaces, and III - Liver postmortem slicing showing fibrosis fascioliasis tunnels. (c) Macroscopic leaf-like *Fasciola spp*.

Grossly regarding fascioliasis infection during slaughterhouse postmortem inspection (Figure-2a) showing the external smooth liver surface declared several white or creamy tunnels ranged from few millimeters to nearly 3 cm (Figure-2b), represented the postmortem liver fibrosis appear from external liver surface. Fascioliasis tunnels which observed from intact liver surfaces oozing grassy blackish hemorrhagic exudate, and declared different took photos of creamy leaf-like *Fasciola* spp. about 1.5-2.0 cm in length and about 1.0 cm in width (Figure-2c).

## Discussion

*Fasciola* spp. is a parasite threating domestic ruminants and public health. Transmission of this trematode infection is depending on the presence of intermediate “lymnaea snail” host and final host. This snail host commonly presents in high density during rainfall period annually and/or in highly moist pastures soil [13,18].

The overall prevalence rate of fascioliasis in the examined cattle and bovine slaughtered in El-Kharga municipal abattoir was about 695/2251 (30.88%) which nearly agreed with Morsy *et. al*. [19], who previously found 25.5% in Egypt. On the other hand, higher incidences of fascioliasis have been recorded by Pfukenyi and Mukaratirwa [20], who reported 37.1% in Zimbabwe and Abraham and Jude [13] recorded 44.8% in Nigeria. However, there were some remarkable lower results reported by Mellau *et al*. [21], who found 16.3% in Tanzania, Haridy *et. al*. [22] noted 21.8% in Gahrbia Governorate, Afrakhosravi [23] reported 11.09% in Iran, and Mungube *et. al*. [24] recorded 26% in Kenya.

Human fascioliasis was been occurred after the consumption of encysted cercaria and not by eating of animal livers infected by adult *Fasciola* spp. the ingestion of watercress vegetables grown along contaminated water by snails and domestic ruminant fecal matters with adult parasite [25].

Our reported seasonal liver fascioliasis condemnation rates revealed that buffaloes fascioliasis is higher during winter and autumn than cattle fascioliasis while vice versa condition reported during spring and summer. The buffaloes rates were (46.15%, 30.77%, 15.38%, and 7.69%) during winter, autumn, spring, and summer, respectively, whereas in case of cattle, it were (35.04%, 23.75%, 22.73%, and 18.48%) during winter, autumn, spring, and summer, respectively. This finding might be attributed to raining season and presence of fresh green grazing pasturing. This finding was supported by the previous findings reported by Adedokun *et al*. [26] who reported in winter (52.3%) and in dry season (21%) in Nigerian cattle, while, fasciolosis was highest in winter (around the raining periods) and/or dampness area due to spreading of the snails host [13,23,27,28].

Fascioliasis occurs mainly not only in children living in rural settings but also in people living in urban areas by metacercarial of the fluke is ingested along with water cress salad and vegetables grown along banks of water reservoirs inhabited by potential snail hosts. About 2.4 million people infected worldwide and 180 million are at risk of the infection fascioliasis commonly asymptomatic children infection with mild anemia. Humans’ fascioliasis is mainly correlated with highly eggs excreted areas and not related with highly animals’ fascioliasis and sometimes infection transmitted by human stool contamination [29].

In this study, the routine macroscopic postmortem fascioliasis inspection revealed that infected liver is very hard may have numerous injuries with congestion, enlargement with very hard fibrosis. Postmortem visually examination of intact liver also showing the presence of different sizes (1.5-2.7 cm) of *Fasciola* spp. impeded on the hepatic tissue with characteristic white or creamy color. Hepatic postmortem incision is showing thick wall fibrosis by fascioliasis tunnels which oozing grassy blackish exudate and debris. The trials to opening this tunnel exerted leaf-like liver flukes that diminished infected liver and carcass value and resulted in rejection of liver by consumers. Similar lesions were observed by authors in Bangladesh [18] and in Nigeria [2,13].

According to Egyptian veterinary authorities, detection of fascioliasis in liver should be removed total liver condemnation or partial affected lobes after performing boiling tests and rapid phase according to parasitic infestation density and extension. The rest carcass was been released for human consumption [25].

Controlling fasciolosis mainly by anthelmintics, which act against mature stages only. Triclabendazole is the only drug, which affects against both immature and mature stages fascioliasis. Anthelmintic administered during December/January and from April/May for controlling chronic fasciolosis, a third dose should be given in August. However, molluscicides were been recommended for snail control [20,30].

## Conclusion and Recommendation

The present study revealed a moderate fasciolosis infestation in cattle in the maniacal abattoir in El-Kharga, New Valley, Egypt, and the study is recommended that the importance to increasing the public health fascioliasis awareness should be taken seriously to enhancing snail and fasciolosis control at farm levels to diminish the economic losses due to infection. Thoroughly meat inspection should also be taken on abattoir.

## Authors’ Contributions

NTE: Corresponding author of the manuscript, study design, collection of the samples, photography, collection of data from the slaughterhouse, drafted and revised the manuscript, and data analysis. WGM: Helped in laboratory examination. Both authors shared laboratory examination and data analysis. Both authors have read and approved the final manuscript.
